# The sequential mediating roles of self-esteem and life satisfaction in the relationship between health perception and perceived social status among college students

**DOI:** 10.3389/fpsyg.2026.1890570

**Published:** 2026-08-26

**Authors:** Eun Bee Kim, Jhong Yun Kim

**Affiliations:** 1 Department of Liberal Arts, Wesley Creative Convergence College, HyupSung University, Hwaseong, Republic of Korea; 2 School of Management, Hanbit University, Peachtree Corners, GA, United States

**Keywords:** college students, health perception, life satisfaction, mediating effect, perceived social status, self-esteem

## Abstract

**Background:**

College students face significant challenges in their physical and psychological well-being, potentially affecting their perception of social status. This study examines the association between health perception and perceived social status among college students and investigates the potential mediating roles of self-esteem and life satisfaction.

**Methods:**

Data from 5,966 participants (50.8% female, 49.2% male) were analyzed from the 2016 Korean Education and Employment Panel Survey (KEEP). Analyses were conducted using SPSS 21.0, AMOS 22.0, and the PROCESS macro. The research model examined the direct relationships between health perception, self-esteem, life satisfaction, and perceived social status, as well as the potential mediating roles of self-esteem and life satisfaction.

**Results:**

Health perception was positively associated with self-esteem (*β* = 0.232, *p* < 0.001), life satisfaction (*β* = 0.273, *p* < 0.001), and perceived social status (β = 0.120, *p* < 0.001). Self-esteem showed positive associations with life satisfaction (*β* = 0.359, *p* < 0.001) and perceived social status (β = 0.078, *p* < 0.001). Life satisfaction was significantly related to perceived social status (*β* = 0.419, *p* < 0.001). The indirect association of health perception with perceived social status through self-esteem was 0.018 (95% CI [0.011, 0.025]), through life satisfaction was 0.115 (95% CI [0.101, 0.129]), and through both mediators sequentially was 0.035 (95% CI [0.029, 0.041]).

**Conclusion:**

Results suggest that college students’ health perception may be associated with their perceived social status both directly and indirectly through potential mediating roles of self-esteem and life satisfaction. Because these findings are based on cross-sectional secondary data, they represent statistical associations rather than confirmed causal mechanisms. Health awareness, self-esteem, and life satisfaction were associated with more positive perceived social status; however, longitudinal research is needed before implications for educational interventions can be drawn.

## Introduction

College students occupy a critical developmental phase characterized by significant transitions in both physical and psychological domains. As they navigate the challenging bridge between adolescence and adulthood, these individuals face unprecedented stressors related to identity formation, academic achievement, career preparation, and social integration. This multifaceted transition has been further complicated by recent global challenges, resulting in a concerning rise in psychological distress among young adults. According to recent analyses by the Health Insurance Review and Assessment Service, individuals in their twenties represent both the largest demographic and fastest-growing population diagnosed with depression and anxiety disorders (Kim, 2022). This alarming trend necessitates a more comprehensive understanding of the complex interrelationships between physical, psychological, and social factors affecting college students’ wellbeing and self-perception.

While policy interventions targeting college students have historically prioritized employment outcomes and economic stability, there remains a critical gap in addressing the multidimensional determinants of student wellbeing, particularly concerning psychological and physical health domains (Han et al., 2017). This study aims to address this gap by examining the interplay between health perception, self-esteem, life satisfaction, and perceived social status among college students.

Perceived social status encompasses both objective indicators (income, education, occupation) and subjective evaluations of one’s relative position within society (Keum and Baek, 2011; Yoon and Kim, 2008; Lee and Yoon, 2006). Previous research has demonstrated a significant relationship between physical health perception and subjective social status (Fors Connolly and Johansson Seva, 2018), suggesting that individuals who view themselves as healthier tend to perceive their social standing more favorably. However, the psychological mechanisms mediating this relationship remain inadequately explored, particularly within college student populations.

Life satisfaction, defined as a subjective evaluation of one’s overall well-being, represents a critical indicator of student adjustment and future trajectory. As Cha (2001) notes, life satisfaction reflects the positive, proactive aspects of an individual’s subjective experience. For college students, life satisfaction not only serves as a barometer for current adaptation but also influences career planning and post-graduation outcomes, thereby warranting substantial scholarly and social attention. Research by Kim (2012) has linked life satisfaction to reduced feelings of alienation and frustration, while international studies indicate that positive life evaluations facilitate broader, more proactive thinking and behavior (Fredrickson, 2001). Additionally, this broadening of thought and behavior has, in turn, been associated with greater subjective vitality (Fredrickson, 2001), further emphasizing its importance as both a present-focused evaluation and future-oriented predictor.

Self-esteem referring to an individual’s self-evaluation regarding their worth and capabilities represents another crucial psychological resource for college students navigating this transitional period. Self-esteem serves as a foundation for overcoming obstacles (Swann et al., 2007) and is particularly susceptible to fluctuation during college years as students negotiate new challenges and identities (Erol and Orth, 2011). Research consistently demonstrates that youth with high life satisfaction also exhibit elevated self-esteem (Gilman and Huebner, 2006), suggesting a reciprocal relationship whereby positive life evaluations enhance self-perceptions and vice versa.

Health perception, encompassing both objective health status and subjective health evaluations, constitutes a fundamental aspect of college students’ overall well-being. Despite being at a life stage characterized by peak physical vitality, students’ perceptions of their health status significantly influence health-related behaviors and attitudes that may persist throughout their lives. Subjective health perception, an individual’s self-assessment of their health status, has been identified as a powerful predictor of life satisfaction (Wu, 2010) and represents one of the most sensitive indicators for measuring age-related health levels (Shin, 2011). Moreover, health perception captures dimensions of health status that cannot be determined solely through medical tests or objective measures (Lee et al., 2011; Berger, 1985), making it an invaluable indicator for both researchers and policy planners.

Despite growing recognition of health perception’s importance, empirical research examining its relationship with perceived social status among college students remains limited, particularly regarding the potential mediating roles of psychological resources such as self-esteem and life satisfaction. Therefore, this study aims to examine the associations between college students’ health perceptions and their perceived social status, with specific attention to the mediating effects of self-esteem and life satisfaction within this relationship. By elucidating these pathways, this research seeks to provide evidence-based insights for developing targeted interventions to enhance college students’ psychological well-being and social integration.

## Review of literature

### Health perception

Health perception encompasses both objective and subjective evaluations of one’s physical and mental well-being. Subjective health perception, in particular, represents an individual’s self-assessment of their health status and serves as a significant predictor of overall well-being (Farmer and Ferraro, 1997; Liang, 1982). Recent studies have highlighted that subjective health perception is more than a simple health indicator—it integrates medical, behavioral, and psychosocial factors and strongly correlates with quality of life and survival likelihood (Nam et al., 2014; Seo and Lee, 2011).

College students represent a unique population with regard to health perceptions. While they typically experience peak physical vitality, they simultaneously face significant stressors that can impact their mental health and overall well-being (Barsell et al., 2018; Freeman et al., 2002). Recent research indicates that college students’ health perceptions may be particularly malleable during this transitional period and significantly influence their health behaviors and future health trajectories (Walker et al., 2025).

The significance of health perception as a construct extends beyond physical health outcomes. Wu (2010) found that health perception correlates more strongly with life satisfaction than almost any other factor, making it a critical predictor of overall well-being. Additionally, health perception represents an individual’s assessment of their health from past to present, encompassing their health trajectory over time (Nam et al., 2014). This comprehensive nature makes health perception particularly valuable for understanding college students’ current and future well-being.

### Self-esteem and life satisfaction

Self-esteem and life satisfaction represent core components of psychological well-being with particular relevance during the college years. Self-esteem, defined as an individual’s evaluation of their worth and abilities, typically undergoes significant fluctuations during emerging adulthood as individuals navigate identity formation and new social environments (Erol and Orth, 2011; Gilman and Huebner, 2006; Wu, 2010; Shin, 2011; Lee et al., 2011; Berger, 1985; Farmer and Ferraro, 1997; Liang, 1982; Nam et al., 2014; Seo and Lee, 2011; Barsell et al., 2018; Freeman et al., 2002; Walker et al., 2025; Harter, 1983). Life satisfaction, which encompasses an individual’s cognitive assessment of their overall life quality, similarly reflects the dynamic nature of college students’ experiences as they progress through academic and personal milestones (Cha, 2001; Lee and Lee, 2015).

The relationship between self-esteem and life satisfaction has been well-established in previous research. Diener and Diener’s (1995) cross-cultural study identified self-esteem as a significant predictor of life satisfaction across various cultural contexts. This relationship has been further corroborated among Korean young adult populations, where positive self-evaluation has been consistently linked to greater overall life satisfaction, suggesting developmental continuity from adolescence into emerging adulthood.

Although conceptually related, self-esteem and life satisfaction represent distinct constructs influenced by different variables (Huebner et al., 1999). Self-esteem focuses specifically on self-evaluation, while life satisfaction encompasses a broader assessment of various life domains, including school, family, and social relationships (Çivitci and Çivitci, 2009). This distinction is particularly relevant for college students navigating multiple life domains simultaneously.

Empirical evidence suggests that self-esteem often precedes life satisfaction in causal pathways. Çivitci and Çivitci (2009) demonstrated that self-esteem functions as a mediating variable that predicts subsequent life satisfaction. This finding aligns with broader theoretical frameworks suggesting that positive self-evaluation contributes to more favorable assessments of one’s overall life circumstances. However, this sequential relationship requires further investigation within the specific context of college students’ development.

### Perceived social status

Perceived social status refers to individuals’ subjective assessment of their position within social hierarchies (Lee, 2021). Unlike objective socioeconomic status (SES), which is typically measured through income, education, and occupation, perceived social status captures individuals’ internalized sense of where they stand relative to others in society (Ostrove et al., 2000). For college students, this perception may be particularly meaningful as they navigate transitions between various social contexts and anticipate future professional roles. It is important to note that perceived social status is still under active development for this population: many students are not yet professionally active, do not hold independent financial resources, and derive much of their current social standing from their parents’ socioeconomic position rather than from achieved status of their own. Perceived social status among students may therefore reflect an anticipatory, aspirational judgment about future standing as much as an evaluation of present circumstances, a nuance that should be considered when interpreting findings involving this population.

Research indicates that perceived social status is influenced by multiple factors beyond objective socioeconomic indicators. Lee (2021) found that occupational prestige, health status, and job satisfaction all contribute to individuals’ assessment of their social standing. This multidimensional nature makes perceived social status a complex construct that integrates various aspects of individuals’ lived experiences and self-concept.

The relationship between health and perceived social status appears bidirectional, with each variable potentially influencing the other over time. Improvements in health may enhance perceived social status through increased social participation and achievement, while higher perceived social status may promote better health through improved access to resources and reduced psychosocial stress (Fors Connolly and Johansson Seva, 2018; Lee, 2021). This dynamic interplay suggests potential value in examining mediating factors that might explain these connections.

Recent research has begun to explore the relationship between perceived social status and psychological well-being among college students specifically. Aeva (2018) found that higher perceived social status correlates with increased life satisfaction in this population, suggesting that perceived social status may represent an important component of overall well-being during the college years. However, the mechanisms connecting health perception to perceived social status among college students remain underexplored, highlighting the need for further investigation.

### Relationships among study variables

While previous research has established connections between individual pairs of variables in our model, fewer studies have examined comprehensive pathways connecting health perception, self-esteem, life satisfaction, and perceived social status. Kim and Park (2014) found that better health status was associated with improved academic achievement motivation and life satisfaction among students, but did not identify mediating variables explaining these relationships. Similarly, Lee (2014) established direct correlations between health status, perception of social status, and self-efficacy in middle-aged adults, but did not examine potential sequential mediating effects.

Multiple theoretical frameworks suggest potential pathways connecting our study variables. Drawing on Hobfoll’s Conservation of Resources (COR) theory, health may constitute a personal resource whose perceived adequacy shapes individuals’ capacity to maintain self-esteem and life satisfaction; resource loss or threat—including perceived poor health—is proposed to undermine psychological well-being, while resource gain may support positive self-evaluation. Complementarily, according to Fredrickson’s (2001) broaden-and-build theory, positive health perceptions may be associated with higher self-esteem by expanding individuals’ cognitive and behavioral repertoires. Higher self-esteem may subsequently relate to greater life satisfaction through more positive evaluations of various life domains (Lyubomirsky et al., 2005). The link from life satisfaction to perceived social status may be further understood through Social Comparison Theory: individuals who feel satisfied with their lives may use more favorable reference points when evaluating their social standing relative to peers, thereby reporting higher perceived social status. Finally, greater life satisfaction may relate to higher perceived social status by enabling individuals to present themselves more positively in social interactions and achieve greater success in various life domains (Çivitci and Çivitci, 2009).

The present study aims to test these theoretical pathways empirically by examining whether self-esteem and life satisfaction sequentially mediate the relationship between college students’ health perception and perceived social status. This investigation addresses significant gaps in existing literature regarding the psychological mechanisms connecting physical well-being to social self-evaluation during this critical developmental period.

## Research methodology

### Research model

**Figure 1:**
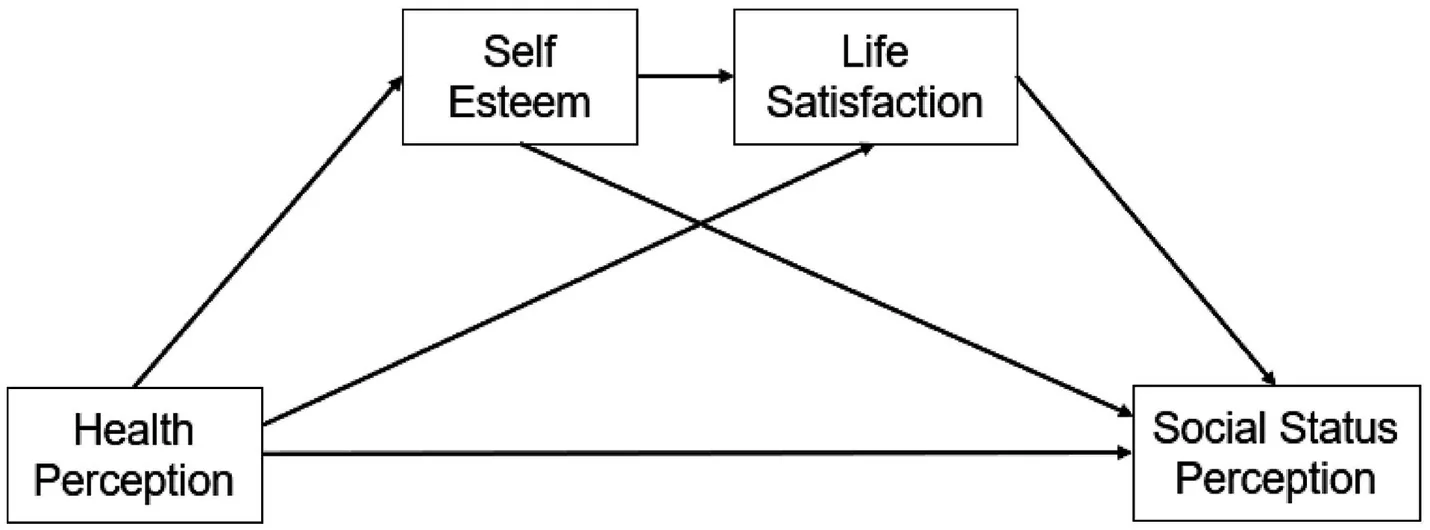
Research model.

The objective of this study was to examine whether self-esteem and life satisfaction statistically mediate the relationship between health perception and perceived social status perception among college students. Based on the literature review, a research model was constructed as shown in Figure 1, proposing that health perception would be associated with perceived social status both directly and indirectly through the potential mediating roles of self-esteem and life satisfaction.

### Data source and participants

This study utilized data from the 2016 Korean Education and Employment Panel Survey (KEEP) conducted by the Korea Research Institute for Vocational Education & Training. The KEEP is a nationally representative longitudinal survey that follows participants for over ten years. The survey has been officially approved by the Korea National Statistical Office (KNSO certificate number: 38902) and follows rigorous methodological protocols to ensure data quality.

The sample for this analysis included 5,966 respondents selected through stratified random sampling to ensure representativeness of the Korean young adult population. Among the participants, 2,938 were men (49.2%) and 3,028 were women (50.8%). Regarding educational background, the highest level of education attained was middle and high school for 4,201 participants (70.4%), community college for 1,044 participants (17.5%), and university for 721 participants (12.1%). In terms of current educational status, 1,726 individuals (28.9%) were attending, on leave from, or had graduated from community college, while 4,240 individuals (71.1%) were attending, on leave from, or had graduated from university (see Table 1).

**Table 1:** Participants.

Categories		Number (percentage %)
Gender	Female	3,028 (50.8)
	Male	2,938 (49.2)
Highest level of education attained	Middle & high school	4,201 (70.4)
	Community college	1,044 (17.5)
	University	721 (12.1)
Current school (attend, leave, and graduate)	Community college	1,726 (28.9)
	University	4,240 (71.1)

To clarify the composition of the analytic sample: “Highest Level of Education Attained” reflects the highest formal degree each respondent had completed at the time of the survey wave, whereas “Current School (Attend, Leave, and Graduate)” reflects respondents’ actual enrollment history in higher education. Because the analytic sample was restricted to the 5,966 respondents who were currently attending, on leave from, or had graduated from a community college or university, every participant had directly experienced tertiary education; the 4,201 respondents (70.4%) whose highest completed degree was middle or high school were, in the large majority, current or former community-college or university students who simply had not yet completed a higher-education degree at the time of data collection, rather than individuals with no tertiary-education experience. Throughout this manuscript, the term “college students” is used to refer to this tertiary-education population, which includes both community-college and university students; the narrower term “university students” is retained only where results are compared by institution type (e.g., Table 2).

**Table 2:** Educational institution differences in study variables.

	Category	Sample	Mean	SD	Levene’s test		t
					F	Sig.	
Health perception	University	4,240	8.50	1.646	2.926	0.087	2.117*
	Community college	1,726	8.40	1.680			
Self esteem	University	4,240	8.65	1.487	24.195	0.000	6.513***
	Community college	1,726	8.37	1.523			
Life satisfaction	University	4,240	7.55	1.649	6.959	0.008	2.702**
	Community college	1,726	7.41	1.756			
Social status	University	4,240	6.34	1.635	0.012	0.914	3.762***
	Community college	1,726	6.17	1.705			

*p < 0.05, **p < 0.01, ***p < 0.001.

### Measures

All measures in this study were derived from the KEEP survey instrument, which has been validated in previous research with Korean young adults. It should be noted that, as with many large-scale nationally representative panel surveys, all four key constructs were measured using single items. Although multi-item scales are generally preferred for their superior psychometric properties, single-item measures are considered acceptable under specific conditions: (1) when the construct is sufficiently concrete and unambiguous, (2) when the research involves secondary analysis of existing panel data where item selection was beyond the researcher’s control, and (3) when the large sample size (*N* = 5,966) mitigates random measurement error through aggregation. Prior research has demonstrated that single-item measures of health perception, life satisfaction, and self-esteem can exhibit adequate convergent validity with multi-item counterparts in large representative samples (Wu, 2010; Cheung and Lucas, 2014; DeSalvo et al., 2006; Robins et al., 2001). Nevertheless, the use of single-item measures precludes confirmatory factor analysis, calculation of composite reliability, and assessment of average variance extracted; findings should therefore be interpreted with appropriate caution. The specific measures used in this study are described below.

#### Health perception

Health perception was measured using a single item from the KEEP panel data: “How do you evaluate your current health perception?” Participants responded on a scale from 1 to 10, with higher scores indicating better perceived health. Single-item measures of health perception have demonstrated adequate reliability and validity in previous research (Wu, 2010; DeSalvo et al., 2006).

#### Self-esteem

Self-esteem was assessed using the item “I believe I’m a good person” from the KEEP panel survey. Participants rated this statement on a scale from 1 to 10, with higher scores indicating greater self-esteem. While multi-item scales are typically preferred for measuring psychological constructs, single-item measures of self-esteem have shown acceptable psychometric properties in large-scale survey research (Robins et al., 2001).

#### Life satisfaction

Life satisfaction was measured using the question “How satisfied are you with your life overall these days?” Participants responded on a scale from 1 to 10, with higher scores indicating greater life satisfaction. Single-item measures of life satisfaction have demonstrated strong correlations with multi-item scales and good test–retest reliability in previous research (Cheung and Lucas, 2014; DeSalvo et al., 2006).

#### Perceived social status

Perceived social status was assessed using the question “How would you rate your current social status?” Participants rated their perceived social status on a scale from 1 to 10, with higher scores indicating higher perceived social status. This approach aligns with established measures of subjective social status used in previous research (Ostrove et al., 2000).

### Data analysis and research procedures

Data analysis was conducted using SPSS 21.0, AMOS 22.0, and Hayes’ PROCESS macro. The analysis proceeded through the following steps.

#### Descriptive statistics

Frequency analysis and descriptive statistics were calculated for all study variables to examine their distributions and central tendencies.

#### Correlation analysis

Pearson correlation coefficients were computed to assess the direction and strength of relationships between health perception, self-esteem, life satisfaction, and perceived social status.

#### Group comparisons

Independent samples t-tests were conducted to examine differences in study variables based on gender and type of educational institution (university versus community college).

#### Direct effects analysis

Multiple regression analyses were performed to examine the direct effects among the study variables following the proposed research model.

#### Mediation analysis

The PROCESS macro (Model 6; Hayes, 2013) was employed to test the sequential mediating effects of self-esteem and life satisfaction in the relationship between health perception and perceived social status. This approach allows for simultaneous testing of direct, indirect, and total effects without requiring separate analytical steps (Lee, 2014). Bootstrap resampling (5,000 samples) was used to generate 95% confidence intervals for the indirect effects, with intervals not containing zero indicating statistically significant mediation (Kim and Park, 2014).

All statistical tests used a significance level of *p* < 0.05. Prior to conducting the analyses, data were screened for missing values and outliers. Missing data were minimal (< 2%) and handled using listwise deletion as recommended for large sample sizes with minimal missing data Prior to the regression and mediation analyses, key regression assumptions were examined. Multicollinearity was assessed using tolerance and variance inflation factor (VIF) values for the predictors of each dependent variable; all tolerance values exceeded 0.70 and all VIF values were below 1.5, well under the conventional cutoff of 10, indicating that multicollinearity was not a concern. Inspection of residuals also indicated no serious violations of the normality or homoscedasticity assumptions (Tabachnick and Fidell, 2013).

## Research results

### Descriptive statistics and correlations

Descriptive statistics and correlations among the study variables are presented in Table 3. The mean scores for health perception (M = 8.47, SD = 1.656) and self-esteem (M = 8.57, SD = 1.503) were relatively high on the 10-point scale. Life satisfaction (M = 7.51, SD = 1.681) was moderately high, while perceived social status (M = 6.29, SD = 1.657) was somewhat lower compared to the other variables.

**Table 3:** Descriptive statistics and correlations among study variables.

Variable	Mean	SD	1	2	3	4
1. Health perception	8.47	1.656	–			
2. Self Esteem	8.57	1.503	0.232*	–		
3. Life Satisfaction	7.51	1.681	0.357*	0.422*	–	
4. Perceived Social Status	6.29	1.657	0.287*	0.282*	0.495*	–

*p < 0.001.

Correlation analysis revealed significant positive relationships among all study variables. Health perception was positively correlated with self-esteem (r = 0.232, *p* < 0.001), life satisfaction (r = 0.357, *p* < 0.001), and perceived social status (r = 0.287, *p* < 0.001). Self-esteem showed positive correlations with life satisfaction (r = 0.422, *p* < 0.001) and perceived social status (r = 0.282, *p* < 0.001). Life satisfaction demonstrated the strongest correlation with perceived social status (r = 0.495, *p* < 0.001). These results indicate that all variables in the model have direct relationships with each other, supporting further investigation of potential mediating pathways.

### Gender and educational institution differences

Independent samples t-tests were conducted to examine differences in the study variables based on gender and type of educational institution. Table 4 presents the results of gender comparisons. Among the four variables, only health perception showed a significant gender difference (t = 13.040, *p* < 0.001), with male college students (M = 8.75, SD = 1.618) reporting better perceived health compared to female college students (M = 8.20, SD = 1.647). No significant gender differences were found for self-esteem, life satisfaction, or perceived social status.

**Table 4:** Gender differences in study variables.

Category	Group	N	Mean	SD	Levene’s F	Levene’s Sig.	t
Health Perception	Male	2,938	8.75	1.618	55.645	0.000	13.040*
	Female	3,028	8.20	1.647			
Self Esteem	Male	2,938	8.57	1.527	3.594	0.058	−0.230
	Female	3,028	8.57	1.479			
Life Satisfaction	Male	2,938	7.55	1.666	0.723	0.395	1.854
	Female	3,028	7.47	1.696			
Social Status	Male	2,938	6.26	1.718	8.942	0.003	−1.310
	Female	3,028	6.32	1.596			

*p < 0.001.

Table 2 presents differences in study variables based on educational institution type. For all four variables, university students reported higher levels compared to community college students, and these differences were statistically significant. Specifically, university students reported better health perception (t = 2.117, *p* < 0.05), higher self-esteem (t = 6.513, *p* < 0.001), greater life satisfaction (t = 2.702, *p* < 0.01), and higher perceived social status (t = 3.762, *p* < 0.001) compared to community college students.

### Direct effects

To examine the direct effects among the study variables, multiple regression analyses were conducted following the proposed research model. The results are presented in Table 5.

**Table 5:** Direct effects among study variables.

Dependant variables	Independent variables	b	β	SD	t	p	LLCI	ULCI
							95%CI	95%CI
Self-esteem	(constant)	6.782		0.099	68.732	0.000	6.589	6.976
	Health perception	0.211	0.232	0.011	18.454	0.000	0.1885	0.2333
	R = 0.232; R^2^ = 0.054; F = 340.540; *p* = 0.000							
Life satisfaction	(constant)	1.715		0.132	13.022	0.000	1.457	1.973
	Health perception	0.278	0.273	0.012	23.697	0.000	0.255	0.301
	Self-esteem	0.402	0.359	0.013	31.101	0.000	0.376	0.427
	R = 0.499; R^2^ = 0.249; F = 989.090; *p* = 0.000							
Perceived social status	(constant)	1.442		0.130	11.06	0.000	1.186	1.698
	Health perception	0.120	0.120	0.012	10.033	0.000	0.097	0.144
	Self-esteem	0.086	0.078	0.014	6.299	0.000	0.059	0.112
	Life satisfaction	0.413	0.419	0.013	32.665	0.000	0.388	0.438
	R = 0.513; R^2^ = 0.264; F = 711.257; *p* = 0.000							

First, the analysis of the direct effect of health perception on self-esteem showed that health perception had a significant positive effect on self-esteem (b = 0.211, *β* = 0.232, *p* < 0.001), explaining 5.4% of the variance in self-esteem (R^2^ = 0.054, *F* = 340.540, *p* < 0.001).

Second, examining the effects on life satisfaction revealed that both health perception (b = 0.278, β = 0.273, *p* < 0.001) and self-esteem (b = 0.402, β = 0.359, *p* < 0.001) had significant positive effects on life satisfaction. Together, these variables explained 24.9% of the variance in life satisfaction (R^2^ = 0.249, *F* = 989.090, *p* < 0.001).

Third, analyzing the effects on perceived social status indicated that health perception (b = 0.120, β = 0.120, *p* < 0.001), self-esteem (b = 0.086, β = 0.078, *p* < 0.001), and life satisfaction (b = 0.413, β = 0.419, *p* < 0.001) all had significant positive effects on perceived social status. The three variables collectively explained 26.4% of the variance in perceived social status (R^2^ = 0.264, *F* = 711.257, *p* < 0.001).

### Mediation analysis

To test the mediating effects of self-esteem and life satisfaction in the relationship between health perception and perceived social status, mediation analysis was conducted using Model 6 of the PROCESS macro. Table 6 presents the results of this analysis.

**Table 6:** Indirect effects of health perception on perceived social status.

Path	Effect	B	Boot SE	LLCI	ULCI
Health perception → Self-Esteem → Perceived Social Status	0.018	0.018	0.004	0.011	0.025
Health perception → Life Satisfaction → Perceived Social Status	0.115	0.115	0.007	0.101	0.129
Health perception → Self-Esteem → Life Satisfaction → Perceived Social Status	0.035	0.035	0.003	0.029	0.041
Total	0.168	0.168	0.009	0.151	0.184

The indirect effect of health perception on perceived social status through self-esteem alone was 0.018 (95% CI [0.011, 0.025]). The indirect effect through life satisfaction alone was 0.115 (95% CI [0.101, 0.129]). The sequential indirect effect through both self-esteem and life satisfaction was 0.035 (95% CI [0.029, 0.041]). All bootstrap confidence intervals excluded zero, indicating statistically significant mediation effects.

The total indirect effect was 0.168 (95% CI [0.151, 0.184]), accounting for 58.2% of the total effect (0.288, 95% CI [0.174, 0.287]). This proportion indicates that more than half of the effect of health perception on perceived social status was mediated through self-esteem and life satisfaction, while the direct effect (0.120, 95% CI [0.097, 0.144]) accounted for the remaining influence.

## Discussion and conclusion

This study investigated the relationship between health perception and perceived social status among college students, with particular focus on the mediating roles of self-esteem and life satisfaction. Using data from 5,966 participants from the 2016 Korean Education and Employment Panel Survey, we found that health perception was associated with perceived social status both directly and indirectly through self-esteem and life satisfaction. These findings provide preliminary insights into potential psychological mechanisms that may connect physical well-being perceptions to social self-evaluation during the critical developmental period of emerging adulthood, though the cross-sectional nature of our data prevents causal inferences.

Our analysis revealed that health perception was positively associated with self-esteem, life satisfaction, and perceived social status. These findings align with previous research suggesting that better perceived health may contribute to more positive self-evaluation and greater life satisfaction (Wu, 2010; Seo and Lee, 2011). The significant direct effect of health perception on perceived social status also supports previous studies indicating that physical health can shape individuals’ perceptions of their social position (Fors Connolly and Johansson Seva, 2018; Lee, 2021).

Self-esteem demonstrated significant positive associations with both life satisfaction and perceived social status. This result corroborates prior research identifying self-esteem as an important predictor of life satisfaction (Diener and Diener, 1995; Hwang et al., 2016) and social self-evaluation (Swann et al., 2007). The significant relationship between self-esteem and perceived social status highlights the importance of positive self-regard in shaping college students’ broader social self-concept.

Life satisfaction showed a strong positive association with perceived social status, emerging as the strongest predictor among the study variables (*β* = 0.419). This finding suggests that college students who report greater overall life satisfaction tend to perceive themselves as occupying higher social positions. This relationship may reflect the comprehensive nature of life satisfaction, which encompasses evaluations across multiple life domains that may collectively contribute to social status assessments.

Perhaps most importantly, our mediation analysis suggested significant indirect effects of health perception on perceived social status through self-esteem alone, life satisfaction alone, and self-esteem and life satisfaction sequentially. The sequential mediation pathway provides empirical support for our theoretical model proposing that health perception may enhance self-esteem, which may subsequently be associated with improved life satisfaction, which in turn relates to higher perceived social status.

The total indirect effect (0.168) accounted for 58.2% of the total effect of health perception on perceived social status (0.288), indicating that most of this relationship operates through psychological mechanisms rather than direct influence. This finding suggests the potential importance of considering psychological mediators when examining connections between physical and social well-being among college students, though replication with longitudinal data is needed to establish temporal precedence.

Our findings contribute to existing literature in several important ways. First, this study extends previous research on health and social status by identifying specific psychological pathways connecting these constructs. While prior studies have established correlations between health and perceived social status (Fors Connolly and Johansson Seva, 2018; Lee, 2021), our research clarifies the mediating roles of self-esteem and life satisfaction in this relationship.

Second, our findings provide empirical support for sequential mediation models in understanding college students’ psychological processes. The significant path from health perception to self-esteem to life satisfaction to perceived social status aligns with theoretical frameworks suggesting cascading effects among these constructs (Fredrickson, 2001; Lyubomirsky et al., 2005). This sequential process illustrates how initial health perceptions may be statistically linked to a chain of psychological associations ultimately relating to social self-evaluation. However, the cross-sectional design limits our ability to establish the temporal sequence of these relationships.

Third, our results expand on existing research regarding self-esteem and life satisfaction by suggesting their potential mediating roles in broader psychological processes. Previous studies have typically examined these constructs as either predictors or outcomes (Diener and Diener, 1995; Çivitci and Çivitci, 2009), but our research demonstrates their function as intermediary mechanisms connecting other psychological phenomena. This finding enriches understanding of how self-esteem and life satisfaction may operate within broader networks of psychological constructs, though longitudinal verification is needed.

Finally, our study contributes to developmental literature on emerging adulthood by highlighting potential interconnections among physical, psychological, and social domains during this life stage. College students’ perceptions of their health appear to shape their broader social self-concept through psychological mechanisms, suggesting the potentially holistic nature of development during this period (Erol and Orth, 2011; Walker et al., 2025), though cultural and contextual factors may limit generalizability.

Several tentative practical implications emerge from these findings; however, given that the direct effect of health perception on perceived social status was small (*β* = 0.120) and that all associations are cross-sectional, these implications should be treated as preliminary hypotheses requiring replication rather than as actionable recommendations. First, college health services might consider implementing programs that not only address physical health concerns but also promote positive health perceptions among students. Second, student counseling centers could potentially benefit from integrating self-esteem enhancement strategies into their interventions, given the possibility that higher self-esteem may be associated with improved life satisfaction and social integration. However, the modest effect sizes observed in this study suggest that such interventions are unlikely to produce large improvements in perceived social status in isolation. Third, academic advisors and student affairs professionals should be aware of the potentially interconnected nature of health, psychological well-being, and social perception when supporting students facing challenges in any of these domains.

Our findings suggest potential relationships among health perception, self-esteem, life satisfaction, and perceived social status among college students in Korea. While the mediation analysis identified self-esteem and life satisfaction as possible intermediary mechanisms, the cross-sectional design and methodological limitations prevent definitive conclusions about causality or practical significance. These preliminary insights may inform future research directions and could potentially contribute to holistic approaches to student development, pending replication with stronger research designs. However, practitioners and policymakers should exercise caution in applying these findings until they are validated through longitudinal research with diverse populations and improved measurement approaches.

The study highlights the complex nature of psychological well-being among university students and suggests the potential value of integrated approaches that consider multiple domains of student experience. While these results provide a foundation for future investigation, substantial additional research is needed to establish the robustness, generalizability, and practical utility of these relationships.

### Limitations and future research directions

This study, despite offering meaningful insights, has several important limitations that warrant caution in interpretation and generalizability of the findings.

First, the cross-sectional design inherently restricts the ability to draw causal conclusions between variables. Although we examined a theoretically grounded mediation model, the cross-sectional nature of the data prevents clear inference of temporal precedence among the variables.

Second, all constructs were assessed using single-item self-report measures, which may suffer from limited reliability and validity compared to validated multi-item scales. Single-item assessments are also vulnerable to transient respondent states or response biases and are insufficient for capturing the multifaceted nature of complex psychological constructs. This increases the likelihood of measurement error and decreases the precision of interpretation.

Third, the sample consisted exclusively of Korean university students, raising concerns about the representativeness and external validity of the findings. Cultural values, educational systems, and sociostructural contexts may shape the psychological processes under investigation, and caution should be exercised when generalizing the findings to other populations.

Fourth, while the study emphasized intrapersonal psychological variables, it did not sufficiently account for contextual and environmental influences on perceived social status—such as household income, parental SES, GPA, employment status, depression, age, and peer networks. The omission of these potentially confounding variables raises the possibility of omitted variable bias, whereby relationships observed among the study variables may partly reflect the influence of unmeasured background factors. Future research should incorporate these covariates into regression models to better isolate the unique associations among health perception, self-esteem, life satisfaction, and perceived social status. The exclusion of these potentially influential variables introduces possible confounding effects and reduces the applicability of the findings to real-world contexts.

Of the covariates listed above, the 2016 KEEP wave used for this analysis does not include measures of parental education, household income, or clinical mental-health status, so these could not be added to the present models; age, gender, academic performance (self-reported grades), and current school type were available and are reported descriptively (Tables 2, 4), but were not yet incorporated as covariates in the mediation model. Future analyses using KEEP waves that include fuller socioeconomic and mental-health indicators should incorporate these variables directly. These available variables (age, gender, academic performance, and school type) were not entered as covariates in the primary mediation model for three reasons: the model was specified to provide a parsimonious test of the theoretically hypothesized sequential pathway among the four focal constructs; the analytic sample was drawn through stratified random sampling from a nationally representative panel, which reduces systematic confounding by such background characteristics; and gender and institution type were already examined separately through group comparisons (Tables 2, 4). Future confirmatory analyses should nonetheless include age, gender, academic performance, and school type as covariates to more fully isolate the unique associations reported here.

Fifth, the data were collected in 2016 and are therefore a decade old at the time of this report. Patterns of health perception, self-esteem, life satisfaction, and perceived social status among young people may have shifted since then in response to changing economic conditions, social media use, and the aftermath of the COVID-19 pandemic. Findings should accordingly be interpreted as reflecting the cohort and period studied, and replication with more recent panel data is needed before the results are generalized to today’s university students.

## Data Availability

The data used in this study are publicly available through the Korea Research Institute for Vocational Education and Training (KRIVET). The 2016 Korean Education and Employment Panel Survey (KEEP) can be accessed at: https://www.krivet.re.kr. This study used publicly available secondary data and did not involve any intervention or identifiable personal data. As such, ethical approval was not required. However, the 2016 KEEP dataset is officially approved and administered by the Korea National Statistical Office (KNSO certificate number: 38902). No personal identifiers were used in the analysis.
